# Significant reductions in human visual gamma frequency by the gaba reuptake inhibitor tiagabine revealed by robust peak frequency estimation

**DOI:** 10.1002/hbm.23283

**Published:** 2016-10-06

**Authors:** Lorenzo Magazzini, Suresh D. Muthukumaraswamy, Anne E. Campbell, Khalid Hamandi, Anne Lingford‐Hughes, Jim F. M. Myers, David J. Nutt, Petroc Sumner, Sue J. Wilson, Krish D. Singh

**Affiliations:** ^1^ Cardiff University Brain Research Imaging Centre, School of Psychology, Cardiff University Cardiff CF24 4HQ United Kingdom; ^2^ School of Pharmacy, Faculty of Medical and Health Sciences, Auckland University Auckland 1123 New Zealand; ^3^ School of Psychology, Faculty of Science, Auckland University Auckland 1123 New Zealand; ^4^ The Epilepsy Unit, University Hospital of Wales Cardiff CF14 4XW United Kingdom; ^5^ Division of Brain Sciences Centre for Neuropsychopharmacology, Imperial College London W12 0NN London United Kingdom

**Keywords:** gamma oscillations, peak frequency, bootstrapping, GABA, tiagabine, magnetoencephalography

## Abstract

The frequency of visual gamma oscillations is determined by both the neuronal excitation–inhibition balance and the time constants of GABAergic processes. The gamma peak frequency has been linked to sensory processing, cognitive function, cortical structure, and may have a genetic contribution. To disentangle the intricate relationship among these factors, accurate and reliable estimates of peak frequency are required. Here, a bootstrapping approach that provides estimates of peak frequency reliability, thereby increasing the robustness of the inferences made on this parameter was developed. The method using both simulated data and real data from two previous pharmacological MEG studies of visual gamma with alcohol and tiagabine was validated. In particular, the study by Muthukumaraswamy et al. [2013a] (Neuropsychopharmacology 38(6):1105–1112), in which GABAergic enhancement by tiagabine had previously demonstrated a null effect on visual gamma oscillations, contrasting with strong evidence from both animal models and very recent human studies was re‐evaluated. After improved peak frequency estimation and additional exclusion of unreliably measured data, it was found that the GABA reuptake inhibitor tiagabine did produce, as predicted, a marked decrease in visual gamma oscillation frequency. This result demonstrates the potential impact of objective approaches to data quality control, and provides additional translational evidence for the mechanisms of GABAergic transmission generating gamma oscillations in humans. *Hum Brain Mapp 37:3882–3896, 2016*. © **2016 Wiley Periodicals, Inc**.

## INTRODUCTION

Synchronization of rhythmic neuronal firing in the gamma range (∼30–90 Hz) is a potential mechanism for information coding in the brain [Buzsáki and Wang, 2012; Fries, 2009]. Pyramidal cell populations synchronized by inhibitory gamma‐aminobutyric acid (GABA)‐ergic interneurons produce intra‐cortical local field potential (LFP) oscillations [Gonzalez‐Burgos and Lewis, 2012], which can be recorded with high consistency between primates and humans [Fries et al., 2008]. Through translational research [Hall et al., 2005], gamma oscillations have been implicated in human sensory and cognitive function, as well as in neuropsychiatric disease [see Bosman et al., 2014; Phillips and Uhlhaas, 2015; Sedley and Cunningham, 2013, for reviews]. In the magnetoencephalographic (MEG) signal, sustained narrow‐band gamma oscillations are generated in visual cortex in response to simple contrast pattern stimuli [Adjamian et al., 2004; Hoogenboom et al., 2006]. These responses arise from the interaction between local excitatory and inhibitory networks, which are believed to shape the amplitude, as well as the peak frequency of gamma oscillations [Bartos et al., 2007; Gonzalez‐Burgos and Lewis, 2012].

The peak frequency of visual gamma responses is modulated by properties of the visual stimulus such as size [Gieselmann and Thiele, 2008; Jia et al., 2013; Ray and Maunsell, 2011; van Pelt and Fries, 2013], contrast [Hadjipapas et al., 2015; Jia et al., 2013; Lowet et al., 2015; Perry et al., 2015; Ray and Maunsell, 2010; Roberts et al., 2013], motion [Friedman‐Hill, 2000; Muthukumaraswamy and Singh, 2013; Swettenham et al., 2009], motion velocity [Gray et al., 1990; Gray and Viana Di Prisco, 1997; Orekhova et al., 2015], eccentricity [van Pelt and Fries, 2013], noise masking [Jia et al., 2013], and cross‐orientation masking [Lima et al., 2010; Perry, 2015]. Across individuals, peak gamma frequency correlates with psychophysical performance in visual discrimination tasks [Dickinson et al., 2015; Edden et al., 2009]. Inter‐individual differences in frequency appear to be strongly genetically determined [van Pelt et al., 2012], though the individual peak frequency decreases with age [Gaetz et al., 2012; Muthukumaraswamy et al., 2010; Robson et al., 2015]. Nevertheless, peak gamma frequency is highly reproducible over shorter time scales, and thus represents a suitable measure for within‐subject designs [Muthukumaraswamy et al., 2010; Swettenham et al., 2009].

In‐vitro and in‐vivo animal studies have demonstrated a dependency of peak gamma frequency on the time constants of GABAergic processes [Bartos et al., 2007; Gonzalez‐Burgos and Lewis, 2012]. In very recent years, a limited number of studies combining MEG with pharmacological modulation of neurotransmission have provided initial compelling evidence for the translation of such models to humans. Reduced frequency of gamma oscillations was observed following administration of alcohol [Campbell et al., 2014] and lorazepam [Lozano‐Soldevilla et al., 2014], drugs which enhance GABAergic transmission through different mechanisms. These findings largely support animal models in which inhibitory post‐synaptic currents (IPSCs) of prolonged duration result in synchronized pyramidal neurons firing at slower rhythms, generating gamma oscillations at lower frequencies and with higher amplitudes [Gonzalez‐Burgos and Lewis, 2012].

However, not all human studies are entirely consistent with the animal literature. For example, the GABA_A_ positive allosteric modulator propofol was found to increase gamma amplitude, but left gamma frequency unchanged [Saxena et al., 2013]. More surprisingly, a recent study reported that neither the amplitude nor the frequency of visual gamma responses were modulated by tiagabine [Muthukumaraswamy et al., 2013a], a drug that prolongs IPSC duration by selectively inhibiting the re‐uptake of GABA from the synapse. To date, the reasons behind such inconsistencies remain unknown.

In the present work, we build upon previous research in which the robustness of oscillatory measures in the gamma range was studied with respect to systematic variations of the stimulus configuration [Muthukumaraswamy and Singh, 2013]. Despite the use of optimally designed experimental paradigms, gamma responses in certain participants can be barely detectable or scarcely quantifiable [Hoogenboom et al., 2006; Muthukumaraswamy et al., 2010]. Therefore, our ability to disentangle the relationship between gamma oscillation frequency and other parameters depends on the accurate and reliable estimation of peak frequency. In particular, the inclusion of weakly estimated parameters can lead to enhanced risk of both spurious findings and false negative results. For this reason, we developed a novel approach to identify poor quality data by means of un‐biased procedures that identify confidence intervals on the parameter estimates via bootstrapping. Firstly, we test the method on simulated data, demonstrating its validity as well as its increased accuracy in peak frequency estimation. Secondly, we test the method on a pharmacological MEG study of visual gamma and alcohol, replicating the drug‐induced reduction in peak gamma frequency [Campbell et al., 2014]. Finally, we apply the method to visual gamma data from the pharmacological MEG study of tiagabine by Muthukumaraswamy et al. [2013a]. Contrary to the null result previously reported, we found that pharmacological enhancement of GABAergic neurotransmission by tiagabine produced a marked decrease in the peak frequency of visual gamma oscillations. The result supports the authors' original predictions [Muthukumaraswamy et al., 2013a], and provides additional translational evidence for the neurophysiological mechanisms generating gamma oscillations in humans [Bartos et al., 2007; Buzsáki and Wang, 2012].

## MATERIALS AND METHODS

### Quality Control Method Validation

#### Data simulation

**Figure 1:**
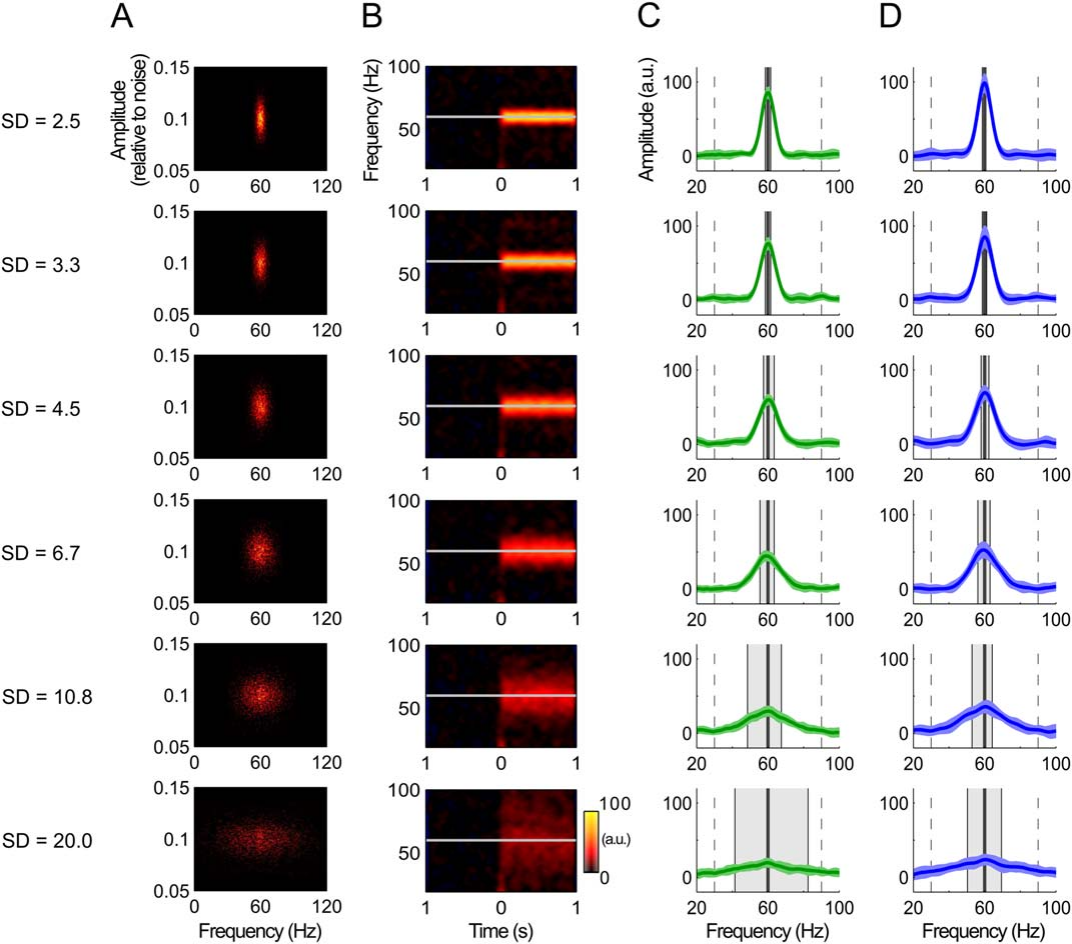
Data simulation. (**A**) Distribution of simulated frequencies and amplitudes pooled across all trials and all datasets, within each of the six noise simulation conditions. Note the decrease in frequency consistency across conditions. (**B)** Time‐frequency spectrograms calculated as percentage change from baseline using the Envelope method, averaged across trials and across datasets. Warm colors index the percentage signal change from baseline, with arbitrary units but the same scale across conditions. The horizontal white line represents the real peak frequency in the data, defined by mean and mode frequency across trials. (**C)** Spectra of percentage signal change from baseline derived using the Envelope method, by averaging across the time dimension (0–1 s) of the time‐frequency spectrograms. Within each condition, the colored shadings represent ± 1 SD across datasets, the thick vertical black lines represent peak gamma frequency, the vertical dashed lines represent the upper and lower limit of the frequency range in which peaks were searched, and the grey background areas define the range of observed peaks across datasets. (**D)** Spectra derived using the Bootstrap method, by averaging the bootstrapped spectra calculated with the smoothed periodogram. The colored shadings, the thick vertical black lines, the vertical dashed lines, and the grey background areas, are the same as in C. It can be noted that the range of peaks estimated with the Bootstrap method (D) was smaller and closer to the real peak frequency of the data, compared with the Envelope method (C). [Color figure can be viewed at http://wileyonlinelibrary.com.]

We used Matlab (The MathWorks) to simulate electrophysiological data, as they would be recorded in visual gamma paradigms with MEG. The data resembled the time course of source‐reconstructed cortical activity in the occipital lobe [see Muthukumaraswamy et al., 2010, for an example with real data], and were generated on a trial‐by‐trial basis (100 trials per dataset). Each trial was composed of noise (2 s), sampled at 1,200 Hz, with 1/*f* frequency scaling of the power spectrum. To reproduce the sustained component of visual gamma responses, the second half of each trial was embedded with a sinusoidal signal (1 s), with different frequency in each trial. The frequency of the oscillation was normally distributed across trials, with both mean frequency and mode frequency of 60 Hz, and standard deviation (SD) of frequency increasing exponentially from 2.5 to 20 Hz across six different conditions (Fig. 1). The six conditions were used to represent the inter‐individual variability in gamma quality that is observed in real participants [e.g., Muthukumaraswamy et al., 2010]. The amplitude of the oscillation was also normally distributed across trials (mean = 10%, SD = 1%, relative to noise amplitude). The phase of the oscillation was generated at random, to avoid phase consistency across trials and reproduce the induced component of visual gamma responses (i.e., time‐locked but not phase‐locked across trials). Thirty datasets were generated in each SD condition. The distribution of frequencies across trials differed slightly between datasets, although it always conformed in mean, mode, and SD, to the appropriate condition. Therefore, by manipulating the consistency of gamma frequency, while precisely controlling for other parameters, we created an ideal scenario for testing the performance of our method with data of progressively degraded quality. The spectra derived with the Envelope and Bootstrap methods are shown in Figure 1C,D, respectively (see section on “Spectral Analysis and Quality Control”). It can be seen that as the SD of the response frequency increased, the range of estimated peak frequencies across datasets (gray background areas in Figure 1C,D) also increased. Overall, the range of peaks estimated with the Bootstrap method was smaller and closer to the real peak frequency of the data, and hence this method was chosen for subsequent analyses.

#### Spectral analysis and quality control

**Figure 2:**
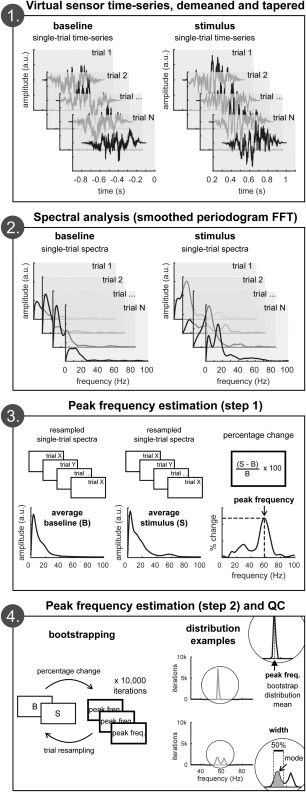
Quality control method. Schematic illustration of the approach to peak frequency estimation and quality control (QC).

An overview of our approach to peak frequency estimation and quality control (QC) is illustrated schematically in Figure 2. To estimate peak gamma frequency, we performed spectral analysis using a Fourier method, the smoothed periodogram [Bloomfield, 2000]. In each trial, the time series of baseline and stimulus (1 s each) were demeaned and tapered with a Hanning window. The raw periodogram was computed separately for baseline and stimulus, and smoothed with a Gaussian kernel (SD = 2 Hz). The single‐trial spectra were averaged across trials, separately for baseline and stimulus, and the amplitude spectrum was calculated as percentage signal change from baseline. In a bootstrap procedure with 10,000 iterations, trials were resampled (with replacement), the resampled single‐trial spectra were averaged, and peak gamma frequency was measured as the spectral peak of greatest increase from baseline, in the 30–90 Hz range. The distribution of peak frequencies across bootstrap iterations was then used in a QC procedure, which evaluated the reliability of the estimated peak frequencies, by calculating the width in frequency that was necessary to accommodate at least 50% of the bootstrapped frequencies around the distribution mode.

The mean of the bootstrapped peak frequencies distribution was used as an improved estimate of peak gamma frequency. To test the validity of this measure, hereafter simply referred to as “Bootstrap peak frequency,” we compared its accuracy to a standard measure used in our lab, the “Envelope peak frequency.” The Envelope peak frequency was calculated by bandpass‐filtering the individual frequencies between 30 and 90 Hz in steps of 0.5 Hz, then calculating the magnitude of the analytic signal (Matlab function: *hilbert)*, to yield the amplitude envelope for this frequency range. The envelopes were baselined, in order to express the response as a percentage change from baseline, and then “stacked” to form a time‐frequency spectrogram (Fig. 1B). From this spectrogram, amplitude was averaged over the stimulus time‐range, within each frequency, yielding the average amplitude spectrum. This spectrum can be used to estimate the peak frequency induced by visual stimulation [see Muthukumaraswamy et al., 2013a, for an example].

### Analysis of Alcohol MEG Data

#### Experimental design and data pre‐processing

We re‐analyzed data from a previous pharmacological MEG study of alcohol [Campbell et al., 2014], using the QC approach outlined above (see section on “Spectral Analysis and Quality Control”). A detailed description of the experimental procedures, including participants, experimental design, MEG acquisition, and data analysis, are reported in Campbell et al. [2014]. Here we provide a brief summary. Sixteen healthy volunteers took part in a single‐blind, placebo‐controlled, crossover study. The study was divided into two days, each consisting of two sessions. Each day began with a “pre‐drink” session, followed by either placebo or alcohol consumption (0.8 g/kg), and by a “post‐drink” session. In each session, MEG was recorded while participants performed the same visual gamma paradigm as described in the tiagabine study (see section on “Experimental Design and Data Pre‐Processing”; [Muthukumaraswamy et al., 2013a]). The only difference between the two visual paradigms was the longer stimulus duration in the alcohol study (1.5 s) compared with the tiagabine study (1 s). For each participant, virtual sensor time series were reconstructed in the voxel of maximal gamma (30–80 Hz) amplitude increase in the occipital lobe.

#### Alcohol data analysis and quality control

Peak gamma frequency reliability estimates and Bootstrap peak frequency measures were obtained using the QC method as described in the validation study (see section on “Spectral Analysis and Quality Control”). The analysis time‐range was −1.4 to −0.1 s for baseline and 0.3–1.5 s for stimulation. The QC criterion was the same as in the tiagabine analysis (∼1.2 Hz; see section on “Tiagabine Data Analysis and Quality Control”). In other words, if 50% or more of the bootstrapped peak frequencies in a given dataset fell within ±1.2 Hz of the bootstrap distribution mode, peak frequency in that dataset was considered reliably estimated. Otherwise, the dataset was marked as of poor quality.

### Analysis of Tiagabine MEG Data

#### Experimental design and data pre‐processing

We re‐analyzed data from a previous pharmacological MEG study of tiagabine [Muthukumaraswamy et al., 2013a], using the QC approach outlined above (see section on “Spectral Analysis and Quality Control”). A detailed description of the experimental procedures, including participants, experimental design, MEG acquisition, and data analysis, are reported in Muthukumaraswamy et al. [2013a]. Here we provide a brief summary. Fifteen healthy volunteers took part in a single‐blind, placebo‐controlled, crossover study. The study was divided into two days, each consisting of four sessions. Each day began with a “pre” measurement session, followed by oral administration of either placebo or tiagabine (15 mg; Gabitril®), and by three “post” measurement sessions at 1, 3, and 5 hours after ingestion. In each session, MEG was recorded while participants performed a visual paradigm known to robustly induce gamma oscillations in primary visual cortex (stationary, maximum contrast, three cycles per degree, square‐wave grating). For each participant, the trial time courses were reconstructed by generating a virtual sensor in the voxel of maximal gamma (30–80 Hz) amplitude increase in the occipital lobe. From this point onward, our analysis departed from that described in Muthukumaraswamy et al. [2013a].

#### Tiagabine data analysis and quality control

Peak gamma frequency reliability estimates and Bootstrap peak frequency measures were obtained using the QC method as described in the validation study (see section on “Spectral Analysis and Quality Control”). As the baseline (−0.8 to −0.1 s) and stimulus (0.3–1.0 s) analysis time‐range had a relatively short length of 700 ms, a relatively broad tapered Tukey window (*α* = 0.25) was chosen in order to reduce spectral leakage whilst preserving as much of the signal as possible. The QC criterion used to calculate a reliability measure was based on the frequency resolution of the periodogram (∼1.2 Hz). In other words, if 50% or more of the bootstrapped peak frequencies in a given dataset fell within ±1.2 Hz of the bootstrap distribution mode, peak frequency in that dataset was considered reliably estimated. Otherwise, the dataset was marked as of poor quality.

## RESULTS

### Method Validation

**Figure 3:**
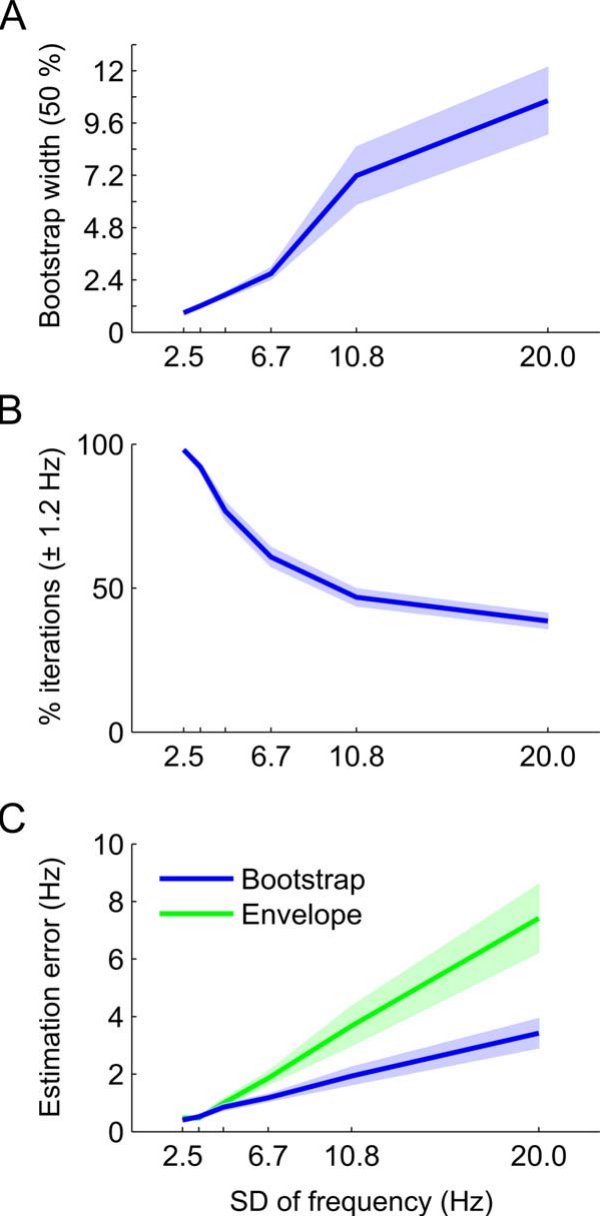
Results of method validation with simulated data. (**A)** Width of the frequency range accommodating 50% or more bootstrapped peaks around the bootstrap distribution mode. (**B)** Percentage of bootstrapped peaks within ±1.2 Hz of the distribution mode. (**C)** Absolute difference between the real and the estimated peak frequencies, averaged across datasets and plotted separately for the Bootstrap (blue) and Envelope (green) estimation methods. In all plots, shaded areas represent ± 1 standard error of the mean (SEM) across datasets. [Color figure can be viewed at http://wileyonlinelibrary.com.]

The QC analysis of the simulated data (see section on “Data Simulation”) showed that, across the six conditions of exponentially decreasing frequency consistency, the width of the Bootstrap peak frequency distribution increased monotonically (Fig. 3A). Likewise, we observed a monotonic decrease in the percentage of bootstrap iterations falling within ±1.2 Hz of the bootstrap distribution mode (Fig. 3B). This indicated the validity of the QC as a method to obtain reliability estimates of peak gamma frequency.

Next, we compared the accuracy in peak frequency estimation between the Bootstrap peak frequency and the Envelope peak frequency. As shown in Figure 3C, the Bootstrap method performed better than the Envelope method, with higher estimation accuracy particularly at the lowest levels of gamma quality.

### Alcohol Data

**Figure 4:**
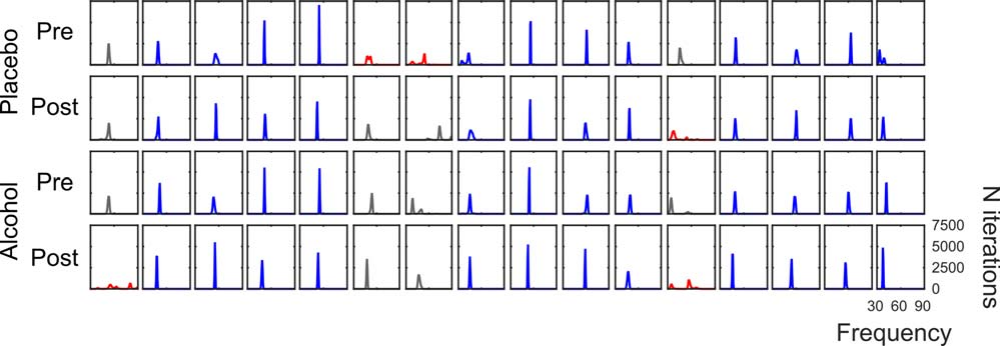
QC results of alcohol data. Distribution of bootstrapped peaks arranged column‐wise by participants, in the placebo (top two rows) and alcohol (bottom two rows) conditions. The QC results are displayed in red (poor datasets) and in blue (good datasets). List‐wise exclusions are displayed in gray. [Color figure can be viewed at http://wileyonlinelibrary.com.]

The QC analysis of the alcohol data (see section on “Alcohol Data Analysis and Quality Control”) revealed that the estimate of peak frequency did not meet our QC reliability criterion in 5 out of 64 datasets (across all participants and conditions; Fig. 4). This resulted in poor quality data in 4 out of 16 participants, as also reported by Campbell et al. [2014].

**Figure 5:**
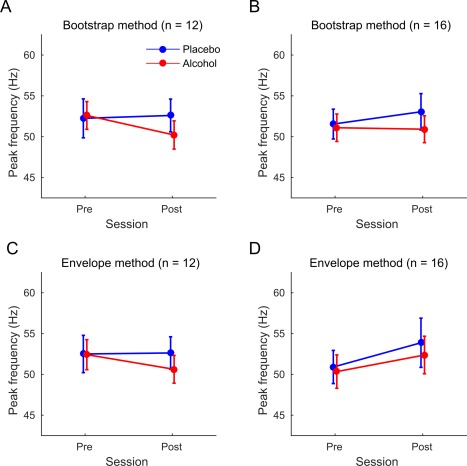
Peak frequency modulations with alcohol. Peak gamma frequency calculated using the Bootstrap method, (**A)** after exclusion of poor quality data, and (**B)** with all participants included. Peak gamma frequency calculated using the Envelope method, (**C)** after exclusion of poor quality data, and (**D)** with all participants included. Peak frequency is plotted in blue for placebo and in red for alcohol. Vertical bars represent ±1 SEM. [Color figure can be viewed at http://wileyonlinelibrary.com.]

To test the effect of alcohol on the frequency of visual gamma, peak frequency was analyzed using a 2 × 2 repeated measures ANOVA, with factors *Drug* (two levels: placebo and alcohol) and *Time* (two levels: pre and post), with the *Drug* × *Time* interaction term being of most interest. To compare how the exclusion of participants and the bootstrap approach to frequency estimation affected the results, the analysis was repeated using the Bootstrap peak frequency (Fig. 5A,B), and the Envelope peak frequency (Fig. 5C,D), with inclusion of good quality data only (Fig. 5A,C), and with all data included (Fig. 5B,D). For reference, the interaction effect observed by Campbell et al. [2014], where peak frequency was estimated in twelve participants using skewed Gaussian function fits, was *F*(1,11) = 13.31, *P* = 0.004.

The Bootstrap method in the 12 accepted participants, illustrated in Figure 5A, resulted in no significant effect of *Drug* (*F*(1,11) = 2.44, *P* = 0.15), a significant effect of *Time* (*F*(1,11) = 5.34, *P* = 0.041), and a significant *Drug* × *Time* interaction effect (*F*(1,11) = 15.58, *P* = 0.002). Peak frequency was significantly reduced by alcohol in the post‐alcohol session compared with both the pre‐alcohol (*t*(11) = −4.68, *P* = 0.001) and the post‐placebo session (*t*(11) = −3.63, *P* = 0.004). There were no significant differences in peak frequency between pre‐ and post‐placebo (*t*(11) = 0.58, *P* = 0.57) or between pre‐placebo and pre‐alcohol (*t*(11) = 0.44, *P* = 0.67). The absence of a significant difference between pre‐ and post‐placebo indicated that the significant main effect of *Time* was driven by the significant difference between pre‐ and post‐alcohol alone.

The results of the Bootstrap method with inclusion of all sixteen participants (Fig. 5B) showed no significant effect of *Drug* (*F*(1,15) = 1.04, *P* = 0.32) or *Time* (*F*(1,15) = 0.31, *P* = 0.59), and no significant *Drug* × *Time* interaction effect (*F*(1,15) = 0.58, *P* = 0.46).

The Envelope method in the twelve accepted participants (Fig. 5C) resulted in no significant effect of *Drug* (*F*(1,11) = 2.51, *P* = 0.14) or *Time* (*F*(1,11) = 2.30, *P* = 0.16), and a significant *Drug* × *Time* interaction effect (*F*(1,11) = 9.16, *P* = 0.012). Peak frequency was significantly reduced by alcohol in the post‐alcohol session compared with both the pre‐alcohol (*t*(11) = −2.57, *P* = 0.026) and the post‐placebo session (*t*(11) = −2.71, *P* = 0.020). There were no significant differences in peak frequency between pre‐ and post‐placebo (*t*(11) = 0.22, *P* = 0.83) or between pre‐placebo and pre‐alcohol (*t*(11) = −0.12, *P* = 0.91).

The results of the Envelope method with inclusion of all 16 participants (Fig. 5D) showed no significant effect of *Drug* (*F*(1,15) = 0.40, *P* = 0.53) or *Time* (*F*(1,15) = 1.26, *P* = 0.30), and no significant *Drug* × *Time* interaction effect (*F*(1,15) = 0.05, *P* = 0.82).

To summarize, using the Bootstrap method to estimate peak gamma frequency and after exclusion of participants based on the QC approach, the interaction effect was replicated at a higher level of significance compared with the Envelope method and the Gaussian function fits. Furthermore, our QC approach resulted in the exclusion of four participants, as also reported by Campbell et al. [2014] based on the absence of a clear peak in at least one of the conditions. No significant interaction was observed, with either the Bootstrap or the Envelope method, when all participants were included in the analysis.

### Tiagabine Data

**Figure 6:**
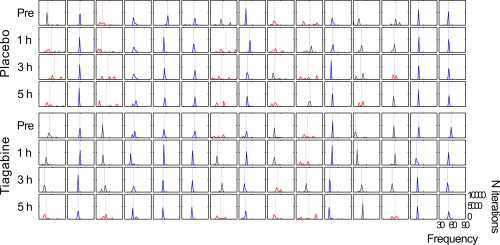
QC results of tiagabine data. Distribution of bootstrapped peaks arranged column‐wise by participants, in the placebo (top four rows) and tiagabine (bottom four rows) conditions. The QC results are displayed in red (poor datasets) and in blue (good datasets). List‐wise exclusions are displayed in gray. The Bootstrap peak frequency (mean of the distribution) is indicated with a vertical line in each dataset. [Color figure can be viewed at http://wileyonlinelibrary.com.]

The QC analysis of the tiagabine data (see section on “Tiagabine Data Analysis and Quality Control”) revealed that the estimate of peak frequency did not meet our QC reliability criterion in 22.5% of the datasets (across all participants and conditions; Fig. 6). Across the eight measurement sessions, the rate of within‐subject data rejection was as high as 62.5% in four participants. Poor quality datasets were treated as missing observations, and excluded from further statistical analysis according to a list‐wise deletion approach.

To compare the spatial distribution of gamma responses between placebo and tiagabine conditions, we averaged the Synthetic Aperture Magnetometry (SAM [Robinson and Vrba, 1999]) beamformer images, separately for accepted and rejected participants. As shown in Supporting Information Figure S1, the SAM images did not show any apparent difference between tiagabine and placebo, or across measurement sessions. However, as expected, the average amplitude of gamma in the occipital cortex appears to be consistently higher in the accepted participants, compared with those whose data was rejected.

**Figure 7:**
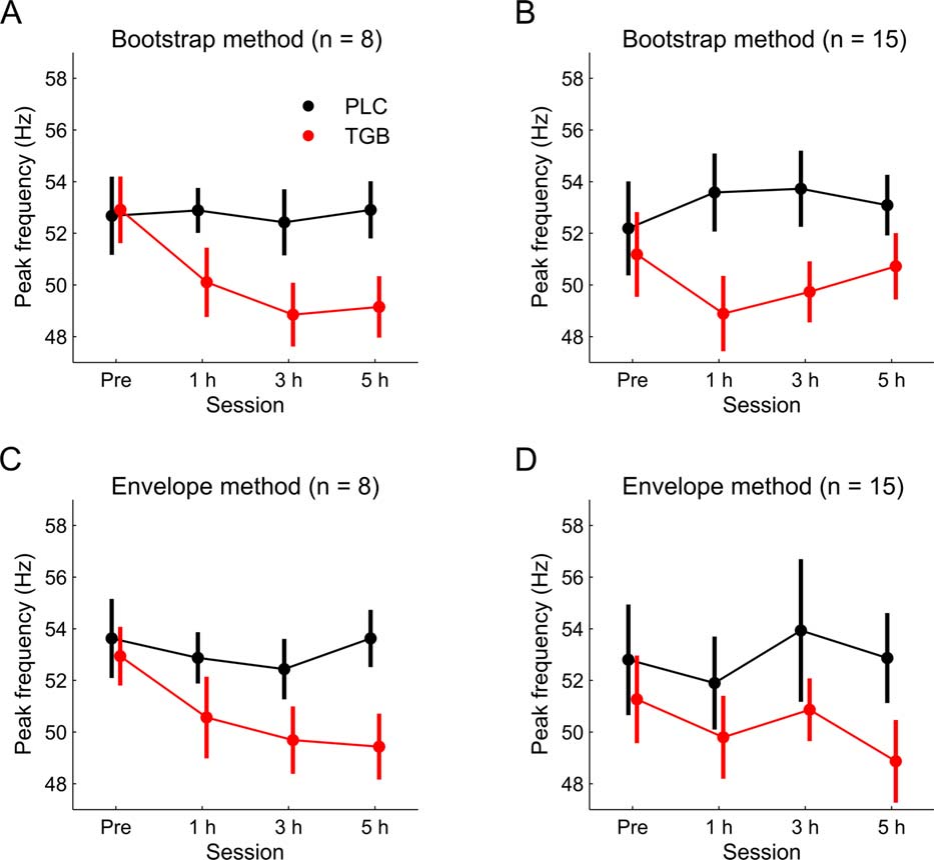
Peak frequency modulations with tiagabine. Peak gamma frequency calculated using the Bootstrap method, (**A)** after exclusion of poor quality data, and (**B)** with all participants included. Peak gamma frequency calculated using the Envelope method, (**C)** after exclusion of poor quality data, and (**D)** with all participants included. Peak frequency is plotted in black for placebo (PLC) and in red for tiagabine (TGB). Vertical bars represent ±1 SEM. [Color figure can be viewed at http://wileyonlinelibrary.com.]

To test our main hypothesis of a shift in the frequency of visual gamma, the Bootstrap peak frequency was analyzed in the eight accepted participants using a 2 × 4 repeated measures ANOVA, with factors *Drug* (two levels: placebo and tiagabine) and *Time* (four levels: pre, 1 h, 3 h, and 5 h). In this analysis design, a significant effect of tiagabine is demonstrated by a significant *Drug* × *Time* interaction. Results, illustrated in Figure 7A, showed a significant effect of *Drug* (*F*(1,7) = 18.8, *P* = 0.003), a marginally non‐significant effect of *Time* (*F*(3,21) = 2.9, *P* = 0.057), and a significant *Drug* × *Time* interaction effect (*F*(3,21) = 3.7, *P* = 0.028).

To investigate the temporal profile of drug modulation, given the significant interaction, we analyzed the simple effects of *Drug* using paired‐sample *t* tests at each of the four time points. There was no difference in peak gamma frequency between the pre‐ingestion sessions of the tiagabine and placebo treatments (*t*(7) = −0.2, *P* = 0.87). In contrast, peak gamma frequency was significantly reduced with tiagabine, compared with the corresponding placebo sessions, at 1 h (*t*(7) = 2.4, *P* = 0.048), at 3 h (*t*(7) = 6.5, *P* = 0.0003), and at 5 h post‐ingestion (*t*(7) = 5.0, *P* = 0.002).

Next, we analyzed the simple effects of *Time* with two 1 × 4 repeated measures ANOVAs, separately for each of the two treatments. There was no effect of time in the placebo treatment (*F*(3,21) = 0.1, *P* = 0.97), suggesting that peak frequency was estimated reliably over repeated sessions under administration of placebo. In contrast, the effect of *Time* was significant in the tiagabine treatment (*F*(3,21) = 7.3, *P* = 0.002). Compared with the pre‐tiagabine session, peak gamma frequency was significantly reduced at 1 h (*t*(7) = 2.6, *P* = 0.036), at 3 h (*t*(7) = 5.2, *P* = 0.001), and at 5 h post‐tiagabine (*t*(7) = 5.5, *P* = 0.001).

**Figure 8:**
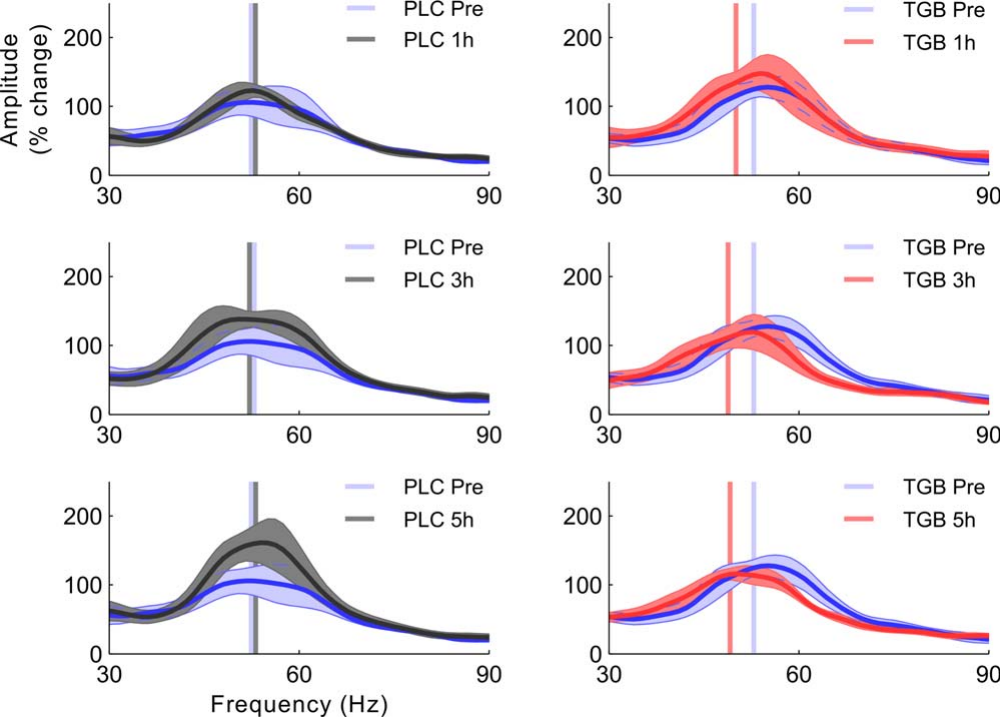
Tiagabine spectra. Amplitude spectra of percentage change from baseline averaged over participants (*n* = 8). Shaded areas represent ±1 SEM across participants. Vertical bars indicate the Bootstrap peak frequency average in the pre‐ (blue) and post‐drug sessions of placebo (PLC; black) and tiagabine (TGB; red). Note that the averaged Bootstrap peaks (vertical lines) appear left of the peak in the averaged spectra because of the averaging procedure, particularly in the right‐hand panels. Specifically, the individual spectra of higher amplitude tended to peak at higher frequencies, resulting in increased amplitude of the portion of the spectrum on the right side of the average peak frequency. [Color figure can be viewed at http://wileyonlinelibrary.com.]

Subsidiary to our main hypothesis, we tested the effect of tiagabine on gamma amplitude with the same statistical analysis used for peak gamma frequency (i.e., a 2 × 4 repeated measures ANOVA in the eight accepted participants). As suggested by visual inspection of the SAM spatial images (Supporting Information Fig. S1), results showed no significant effect of *Drug* (*F*(1,7) = 1.7, *P* = 0.24), or *Time* (*F*(3,21) = 1.5, *P* = 0.24), and no significant *Drug* × *Time* interaction (*F*(3,21) = 2.6, *P* = 0.08). The amplitude spectra of percentage change from baseline in the gamma range are illustrated in Figure 8, averaged across participants. The correspondence between the Bootstrap peak frequencies and the peaks in the gamma range of the raw spectra, across all participant and conditions, is illustrated in Supporting Information Figure S2.

Overall, these results indicated that peak gamma frequency was significantly reduced by tiagabine at each of the three time points measured after drug administration, whereas gamma amplitude was not affected. Furthermore, peak frequency did not differ statistically across the four measurements in the placebo conditions, or between the pre‐tiagabine and the pre‐placebo sessions. Therefore, peak frequency was estimated reliably both at repeated intervals of 2 h, and between sessions as far as 1 week apart. For comparison, results are shown in Figure 7 after the analysis was repeated using the Bootstrap peak frequency (Fig. 7A,B), and the Envelope peak frequency (Fig. 7C,D), with inclusion of good quality data only (Fig. 7A,C), and with all data included (Fig. 7B,D). Despite the session of maximal decrease in frequency differed among the four combinations of method and sample used, the pattern of results appeared qualitatively comparable, apart from when the Envelope method was used with inclusion of all participants.

Additionally, we asked the question of whether differences in gamma quality could be related to differences in the individual peak gamma frequency, across participants. The latter measure has been proposed as an index of local GABA concentration [Muthukumaraswamy et al., 2009] and GABA_A_ receptor density [Kujala et al., 2015], two factors that could potentially influence the variability of our peak frequency reliability estimates. We used Pearson's *r* coefficient to correlate peak gamma frequency, in each experimental session, with peak frequency reliability, as measured by the percentage of bootstrap iterations within ±1.2 Hz of the bootstrap distribution mode. As illustrated in Supporting Information Figure S3A, we found no evidence for a consistent relationship between these two measures, suggesting that the estimated reliability of peak gamma frequency does not depend on the frequency at which gamma peaks occur.

Finally, we investigated the relationship between the magnitude of the change in peak frequency and gamma quality. We first calculated the change in peak frequency by subtracting the pre‐placebo (or pre‐tiagabine) peak frequency from each of the post‐placebo (or post‐tiagabine) sessions, and then correlated this measure with peak frequency reliability. For this purpose, the percentage of bootstrap iterations in the “pre” and “post” sessions were averaged, separately for each correlation. Once again, we found no significant correlations between peak frequency reliability and drug‐induced change in peak frequency. The correlation at 1 h post‐tiagabine was the only positive correlation, and its uncorrected p‐value approached statistical significance (*r* = 0.48, *P* = 0.067). As illustrated in Supporting Information Figure S3B (bottom row, first plot from the left) participants whose peak gamma frequency decreased the most tended to show the lowest reliability estimates, at 1 h post‐tiagabine. This would explain why, after participant exclusion, the greatest reduction in peak frequency was observed in the session at 3 h, rather than 1 h after tiagabine (cf. Fig. 7A,B).

## DISCUSSION

The true nature of neuronal oscillations in the gamma frequency range has been long disputed in neuroscience [Brunet et al., 2014b]. Correspondingly, the choice of spectral method for the analysis of electrophysiological data has also been highly debated [Bruns, 2004; Le Van Quyen and Bragin, 2007; Le Van Quyen et al., 2001; van Vugt et al., 2007]. In the first part of this work, we developed a method based on bootstrapping across trials, which served two purposes. Firstly, we used a measure of spread in the distribution of bootstrapped peaks to estimate the reliability of peak gamma frequency, which in turn allowed the identification of poorly estimated data. Secondly, we measured peak gamma frequency by averaging across the bootstrapped samples, and demonstrated the increased robustness of this measure relative to a more conventional alternative method. In the second part, we used the QC method to re‐analyze data from two pharmacological MEG studies, one in which alcohol was demonstrated to produce a marked decrease in the peak frequency of visual gamma oscillations [Campbell et al., 2014], and one in which tiagabine was reported to modulate stimulus‐evoked responses, but to have no effect on neuronal oscillatory dynamics [Muthukumaraswamy et al., 2013a].

### Methodological Considerations

The QC approach presented here should be considered as a framework for objective quantification of data quality. This neither establishes fixed criteria, nor provides rigid guidelines for data exclusion. Rather, by characterizing data quality with descriptive measures, the method can be used to define explicit exclusion criteria based on unambiguous thresholds, and could prove useful for comparing data across sites, studies, and designs. The Bootstrap peak frequency, calculated from the bootstrap distribution, resulted in an optimal measure when inter‐trial frequency consistency was low in our method validation. However, it is possible that other methods, such as the Envelope approach, could perform with better accuracy under different circumstances, such as when oscillation frequency cannot be assumed stationary. Furthermore, future work on either simulated data or large‐sample datasets will have the opportunity to optimize the current method by using a systematic approach to vary the parameters of each method (e.g., spectral estimation, tapering, number of iterations and jackknife resampling, bootstrap width criterion, etc.) and test how each of these changes impacts on the sensitivity and specificity of the QC results. In particular, sensitivity and specificity can be affected by the width criterion, which determines the threshold for data exclusion. If a strict criterion is chosen, good but not perfect frequency estimates could be inappropriately excluded, potentially leading to a loss of statistical power. On the contrary, the choice of a loose criterion could lead to inclusion of poor estimates and, for example, increased likelihood of false negative results. While the criterion was chosen arbitrarily in this work, with its adequacy being established by visual inspection of the bootstrap distributions, the use of empirically determined criteria in the future would be desirable.

In the second part of this work, the alcohol data by Campbell et al. [2014] was considered as a benchmark to test the validity of our approach on real data. In the original publication, the peak frequency and amplitude parameters were estimated by fitting skewed Gaussian functions to the gamma range of the power spectra. In our analysis, the success of the QC approach was demonstrated in two ways. Firstly, it revealed the presence of poor quality data in the same number of participants as originally reported by the authors, who blind‐screened the data for low‐amplitude gamma responses with no clear peak [Campbell et al., 2014]. Secondly, it replicated the drug‐induced modulations, consisting of a decrease in the peak frequency of visual gamma with alcohol. Together, the results of our validation study on simulated data and our replication of the findings by Campbell et al. [2014] supported the validity of our method to fulfill two purposes; firstly, to identify reliably measured data in the study by Muthukumaraswamy et al. [2013a] and, secondly, to re‐test the effect of tiagabine using optimal estimates of peak gamma frequency.

It should be noticed that, by applying a standard analysis pipeline and avoiding participant exclusion, Muthukumaraswamy et al. [2013a] adopted the least biased approach possible. On the one hand, the rejection of complete datasets is particularly questionable when each individual represents a precious or rare observation and the sample size cannot be readily increased. On the other hand, however, our proposed approach offers the advantage of basing statistical inference on reliably estimated frequency measures. Furthermore, it circumvents the limits of setting simple rejection criteria based on the amplitude of the response, which would show a biased tendency to remove sources of interesting variance, such as drug‐induced reductions in amplitude or disease‐related impairments of oscillatory rhythms.

Of course, rejecting datasets based on reliability of peak frequency estimation is still dependent on signal‐to‐noise and hence amplitude (although amplitude and frequency have been shown to be at least in part dissociated [see Jia et al., 2013]), but not as strongly as if amplitude criteria were used. Put another way, any QC approach is vulnerable to low generalizability of results, as the frequency reduction induced by tiagabine can be demonstrated only for those participants who showed gamma responses of high consistency across trials. Despite the comparability of results illustrated in Figure 7A,B, gamma measures must be of sufficient quality in order for peak frequency modulations to be statistically significant. Results, instead, cannot be generalized to individuals who showed high inter‐trial variability in their response frequency. The factors underlying such differences in the variability of the gamma response frequency are largely unexplored, and remain an open question for future research.

### Peak Gamma Frequency Reduction by Tiagabine

After identification and exclusion of datasets that yielded unreliable estimates of peak gamma frequency, we observed a marked tiagabine‐induced reduction in visual gamma frequency. Peak frequency appeared to be significantly reduced both at 1 h and at 3 h after oral administration (see section on “Tiagabine Data”), in line with the pharmacokinetics of tiagabine showing maximum plasma concentrations occurring between 45 and 150 min after drug ingestion [Leach and Brodie, 1998; Murphy, 2011; Snel et al., 1997]. The average decrease in frequency induced by tiagabine, measured with the Bootstrap method (Fig. 7A) relative to a pre‐tiagabine peak frequency of 52.9 ± 5.0 Hz (mean ± SD across participants), was 2.8, 4.1, and 3.8 Hz at 1, 3, and 5 h, respectively. Interestingly, a comparable effect was observed with alcohol, with peak frequency being reduced on average by 2.4 Hz at less than 1 h after drug consumption, relative to a pre‐alcohol peak frequency of 52.6 ± 5.9 Hz (Fig. 5A).

Overall, these novel tiagabine results are strongly supported by animals models, which demonstrate a close dependency of gamma frequency on the time constants of GABAergic inhibition [Faulkner et al., 1998; Oke et al., 2010; Traub et al., 1996; Whittington et al., 1995, 1996; Xing et al., 2012]. In relatively simple models, the generative mechanisms of gamma oscillations consist of pyramidal cells firing synchronously under the inhibitory control of GABAergic interneurons [see Bartos et al., 2007; Buzsáki and Wang, 2012; Gonzalez‐Burgos and Lewis, 2012; Tiesinga and Sejnowski, 2009, for reviews]. At the synaptic level, tiagabine exerts its effects by selectively inhibiting GAT‐1, the most abundantly expressed GABA transporter (GAT) in the cerebral cortex [Borden et al., 1994; Conti et al., 2004]. By blocking the reuptake of GABA from the synapse, tiagabine elevates the synaptic concentrations of GABA [Dalby, 2000; Fink‐Jensen et al., 1992] and increases the duration of the GABA_A_ receptor‐induced IPSCs [Roepstorff and Lambert, 1994; Thompson and Gähwiler, 1992]. Thus, IPSCs of prolonged duration result in synchronization of neuronal firing at slower rhythms, which in turn translates to LFP oscillations at lower gamma frequencies.

### Relationship Between Gamma and GABA

In non‐invasive human studies, the use of magnetic resonance spectroscopy (MRS) to measure the relationship between GABA and gamma frequency has produced controversial results [cf. Cousijn et al., 2014; Muthukumaraswamy et al., 2009]. Invigorating this debate, a very recent flumazenil‐positron emission tomography (PET) study demonstrated a positive correlation between the frequency of visually induced gamma oscillations and the density of GABA_A_ receptors in early visual areas [Kujala et al., 2015]. Further contribution to the translation of animal models to humans has come from studies combining MEG, to record cortical activity, with the use of pharmacological agents, to modulate neurotransmission [Hall et al., 2010; Muthukumaraswamy, 2014]. Decreased visual gamma frequency in humans was observed after administration of alcohol, which affects GABA and N‐Methyl‐D‐aspartate (NMDA) receptor activity [Campbell et al., 2014], and lorazepam, a positive allosteric GABA_A_ modulator [Lozano‐Soldevilla et al., 2014]. More recently, comparable results were obtained with the NMDA receptor antagonist ketamine [Shaw et al., 2015]. In addition to the frequency modulation, these studies found increased amplitude of gamma responses with GABAergic enhancement, replicating previous results obtained with administration of the GABA_A_ agonist propofol [Saxena et al., 2013]. Increased gamma amplitude accompanying a shift toward lower gamma frequencies may be related to the recruitment of larger pyramidal cell populations achieved under longer periods of inhibition [Gonzalez‐Burgos and Lewis, 2012].

In the current study, however, no significant effects were observed when, subsidiary to our main hypothesis, gamma amplitude was tested with the same analysis used for peak frequency. This could suggest that tiagabine has a specific effect on oscillation frequency via modulation of inhibitory time constants, while leaving other network parameters unaltered. In support of this, animal studies have demonstrated that the duration of IPSCs is prolonged by tiagabine, but IPSC amplitude is not increased [Roepstorff and Lambert, 1994; Thompson and Gähwiler, 1992]. Alternatively, the absence of an effect of drug on gamma amplitude might be explained by lack of sensitivity of amplitude measures themselves. Compared with gamma frequency, gamma amplitude could be more vulnerable to noise, particularly when differences in head movement or head distance from the sensor array in repeated recording sessions are not explicitly controlled for.

### Relationship between Gamma Frequency and Other Parameters

A partially unresolved question is whether the changes in frequency associated with GABAergic neurotransmission are unique to gamma oscillations, or extend to other frequency ranges. Likewise, it is unclear whether the GABAergic influences on oscillatory dynamics are specific to visual areas or extend to other cortices. In sensorimotor regions, administration of a benzodiazepine GABA_A_ positive allosteric modulator produced an alteration of the beta rhythm consisting of decreased frequency and increased amplitude [Jensen et al., 2005]. In other studies, no differences in gamma frequency were observed over motor regions using alcohol [Campbell et al., 2014], lorazepam [Lozano‐Soldevilla et al., 2014], ketamine [Shaw et al., 2015], or tiagabine [Muthukumaraswamy et al., 2013b]. Overall, therefore, the functional significance of shifts in oscillation frequency remains a subject of significant interest.

The frequency of gamma oscillations has been previously related to differences in behavioral performance [Dickinson et al., 2015; Edden et al., 2009], and in cognitive traits of possible clinical relevance [Dickinson et al., 2015; Kahlbrock et al., 2012]. For example, a recent study showed that the normal velocity‐dependent modulation of visual gamma frequency appeared to be impaired in children with autism spectrum disorders [Stroganova et al., 2015]. Although inter‐individual differences in visual gamma frequency have been related to the structural properties of visual cortical areas [e.g., Muthukumaraswamy et al., 2010; Schwarzkopf et al., 2012], other studies do not show a clear dependence [c.f. Kujala et al., 2015; Robson et al., 2015]. Gamma frequency in visual cortex is modulated by sensory input strength, increasing monotonically with respect to stimulus contrast [Perry, 2015; Perry et al., 2015; Ray and Maunsell, 2010]. Increased peak gamma frequency has been reported also for stimuli of smaller size, in both LFP [Gieselmann and Thiele, 2008; Ray and Maunsell, 2011] and MEG recordings [van Pelt and Fries, 2013; although, see Perry et al., 2013 for inconsistent results]. This could be explained with smaller stimuli being represented by smaller neuronal ensembles, which in turn could be synchronized at a higher frequency over a shorter cortical distance [Gieselmann and Thiele, 2008]. Interestingly, gamma responses in monkey visual areas are induced at higher frequencies in response to repeated stimulus presentations compared with novel stimuli [Brunet et al., 2014a], and functionally synchronous networks appear to be tuned to higher frequencies when representing stimuli that are under the focus of attention [Bosman et al., 2012; see also Fries et al., 2001; Fries, 2015]. However, attentional modulation of narrow‐band gamma frequency has not been observed with MEG [e.g., Koelewijn et al., 2013], perhaps due to the different sensitivity of MEG compared with LFPs.

## CONCLUSIONS

The work presented here highlights the potential impact of objective data quality quantification and paves the way for future methodological developments in this direction. Using a novel approach to peak frequency estimation, we demonstrated a reduction in gamma frequency by tiagabine, in those participants with reliable peak frequency estimates. The result is supported by animal models, and provides additional translational evidence of the GABAergic mechanisms generating gamma oscillations in humans.

## Supporting information

Supporting Information Figures.
