# Supplementary material for: Significant reductions in human visual gamma frequency by the gaba reuptake inhibitor tiagabine revealed by robust peak frequency estimation
Source: Hum Brain Mapp. 2016 Oct 6;37(11):3882–96. doi: 10.1002/hbm.23283 (PMC5082569; doi:10.1002/hbm.23283)
Supplement: Supplementary file 1 — Supporting Information Figures. [file HBM-37-3882-s001.docx]

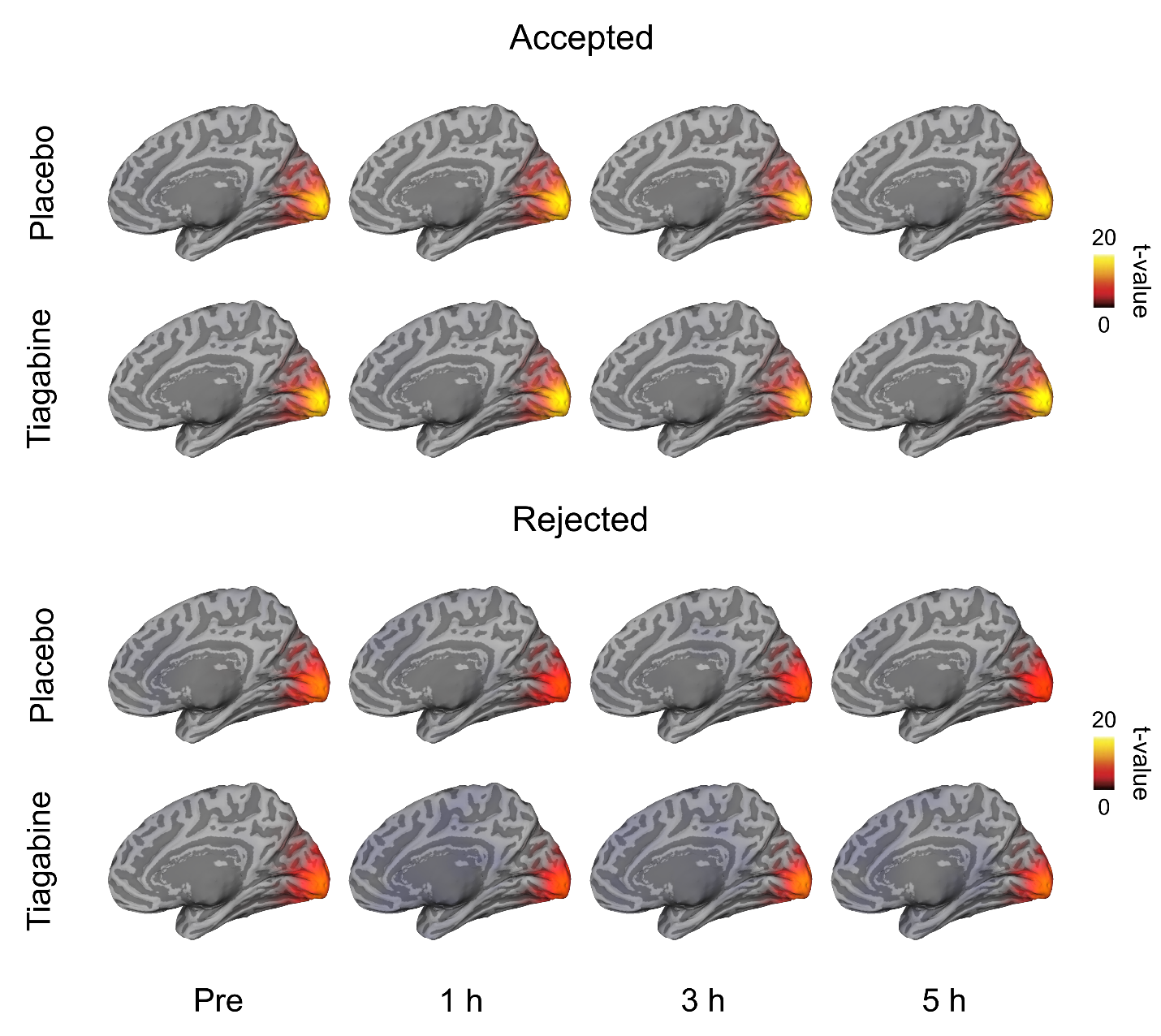


**Supplementary Figure S1. SAM spatial images of tiagabine.**

SAM images contrasting gamma power (30-80 Hz) between baseline (-1 to 0 s) and stimulus (0 to 1 s), at 4 mm isotropic voxel resolution [see Muthukumaraswamy et al., 2013a for methodological details]. The *t*-statistic values were averaged, within each condition, separately for accepted and rejected participants (eight and seven, respectively) and displayed on the right hemisphere of an inflated gray-matter cortical mesh reconstruction of a template-space MRI volume.


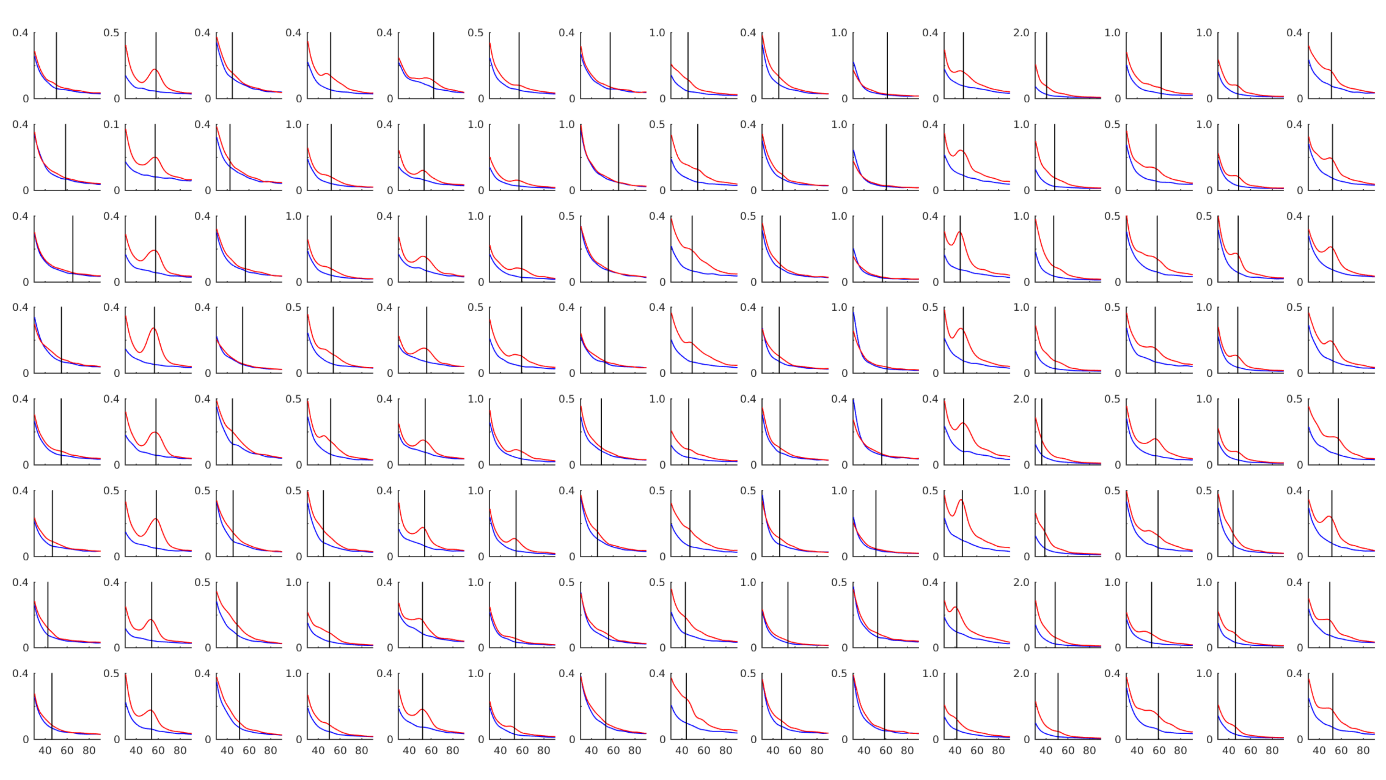


**Supplementary Figure S2. Tiagabine raw spectra.**

Amplitude spectra of baseline (in blue) and stimulus (in red) in the gamma range (30-90 Hz), arranged column-wise by participants, in the placebo (top four rows) and tiagabine (bottom four rows) conditions. The Bootstrap peak frequency is indicated with a vertical line in each dataset.


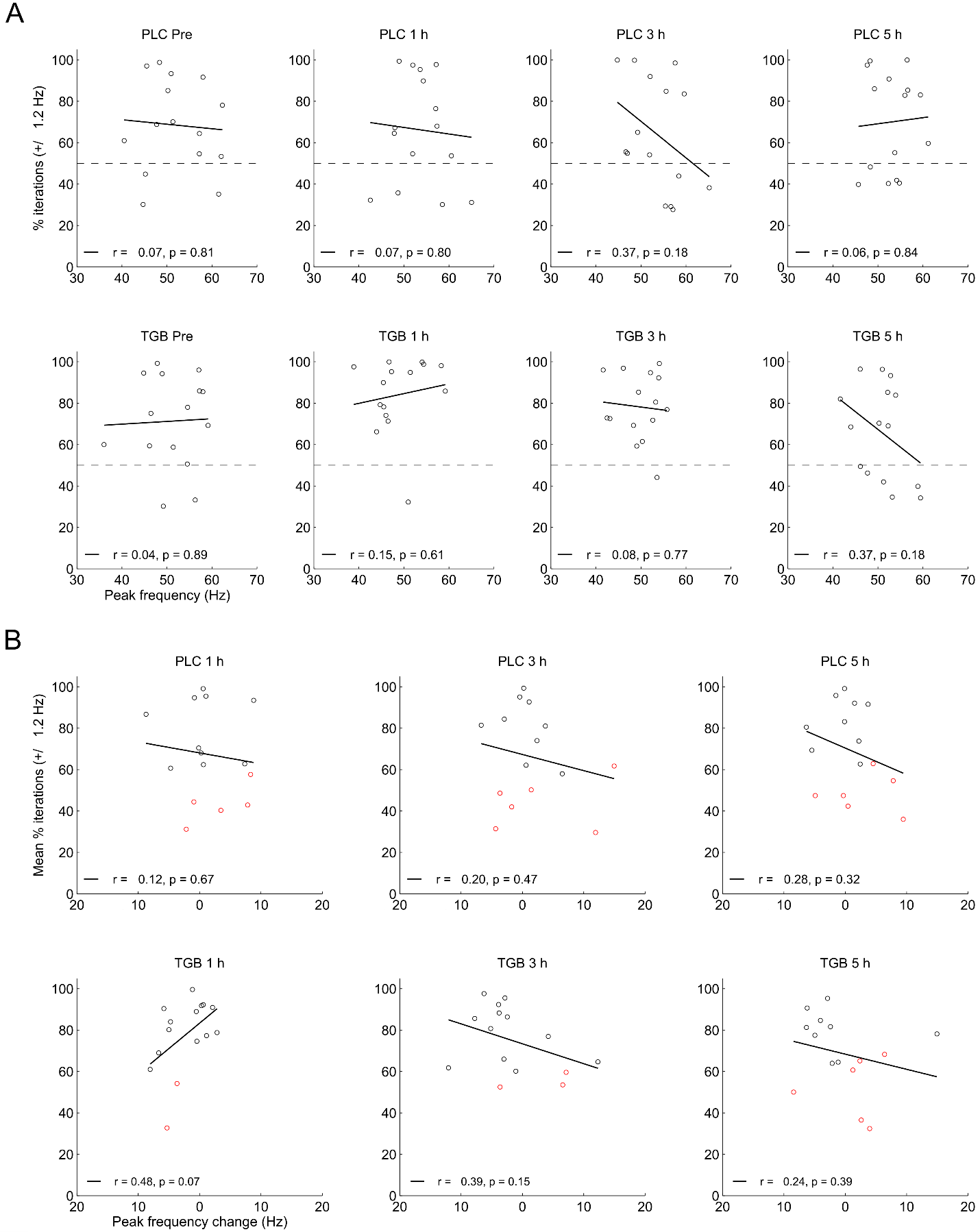


**Supplementary Figure S3. Peak frequency correlations.**

**A)** Correlations between peak gamma frequency and peak frequency reliability, as measured by the percentage of bootstrap iterations within ± 1.2 Hz of the bootstrap distribution mode. In each plot, the horizontal dashed line illustrates the QC criterion, thus observations below the line represent poor peak frequency estimates. **B)** Correlations between the change in peak frequency, calculated by subtracting peak frequency in the ‘pre’ session from each of the ‘post’ sessions, and mean peak frequency reliability, averaged between the ‘pre’ and ‘post’ sessions. Circles plotted in red represent participants with poor peak frequency estimates in either one or both of the ‘pre’ and ‘post’ sessions being correlated.
